# Fabrication, Corrosion, and Mechanical Properties of Magnetron Sputtered Cu–Zr–Al Metallic Glass Thin Film

**DOI:** 10.3390/ma12244147

**Published:** 2019-12-11

**Authors:** Xianshun Wei, Chengxi Ying, Jing Wu, Haoran Jiang, Biao Yan, Jun Shen

**Affiliations:** 1 School of Materials Science and Engineering, Tongji University, 4800 Caoan Road, Shanghai 201804, China; 1730620@tongji.edu.cn (C.Y.); 1610434@tongji.edu.cn (H.J.); yan_biao@tongji.edu.cn (B.Y.); junshen@szu.edu.cn (J.S.); 2 Shanghai Key Laboratory for R&D and Application of Metallic Functional Materials, Tongji University, Shanghai 201804, China; 3 SUSTech Cryo-EM Facility Center, Southern University of Science and Technology, Shenzhen 518055, China; wujinguob@163.com; 4 College of Mechatronics and Control Engineering, Shenzhen University, Shenzhen 518060, China

**Keywords:** thin film metallic glasses, DC magnetron sputtering, nanoindentation, corrosion resistance

## Abstract

The appearance of thin film metallic glasses (TFMGs) is gaining increasing interest because of their unique mechanical and anticorrosion properties and potential engineering applications. In this study, Cu–Zr–Al ternary thin film metallic glasses were fabricated by using DC magnetron sputtering equipment with various target powers. The evolution of the structure was systematically investigated by grazing incidence X-ray diffractometer, scanning electron microscopy, and transmission electron microscopy. The deposition rate increases with the increasing of applied target power. The as-deposited thin films show an amorphous structure. The compositional fluctuations on the nanometer scale indicate the presence of two Cu- and Zr-rich amorphous phases. The electrochemical corrosion measurements indicated that Cu–Zr–Al thin film metallic glasses had good corrosion resistance in the sulfuric acid solution. Nanoindentation results showed that the mechanical deformation was found to be homogenous and reproducible with a high value range for the hardness and modulus.

## 1. Introduction

Metallic glasses are promising materials with wide-ranging applications because of their unique properties, including high strength and toughness, large elastic limits, good soft magnetic, and brilliant corrosion resistance. These features are attributed to the lack of long-range order atomic structure and non-grain boundary effect [1,2,3,4]. This material is attracting increasing research interest since the first metallic glass material was discovered through rapid quenching by Duwez et al. in the 1960s [5]. Many researchers are developing different systems with focus on maximizing the metallic glass sizes, such as Cu- [6,7,8], Zr- [9], and Fe-based [10,11,12] alloy systems. Most metallic glass material exhibits one metallic glass phase, whereas some systems, such as Zr-Ti-Cu-Ni-Be or (Zr, La) -Al-Cu-Ni bulk metallic glass (BMG) systems, exhibit two metallic glass phases due to the phase separation phenomenon [13,14]. The microstructure of these two metallic glass phases provides an opportunity for developing new materials with unique properties. Even though metallic glass material has been comprehensively studied, the development of this material is blocked by the expensiveness, limit, and cooling rate of its complex synthesis with high expense [15,16], thus restricting the application in engineering. In recent years, the appearance of thin film metallic glasses (TFMGs) has drawn increasing attention, and some systems with various compositions showing great glass-forming ability (GFA) and superior properties have been comprehensively studied [17,18,19,20]. At present, thin film metallic glass has been used in many fields particularly in microelectromechanical and nanoelectromechanical devices [21], biochemical, biomedical applications [22,23], diffusion barrier layer [24], alloy coatings [25,26], and nanoimprint technology [27].

The most common method to fabricate the thin film metallic glass is magnetron sputtering, which is known for its high cooling rate, high quality, and good thin film adhesion [28]. The basic sputtering technique contains the pulsed magnetron sputtering process and the direct current (DC) magnetron sputtering [29,30,31]. In previous experiments, element targets were mostly used for co-deposition and adjusting the angles of targets to control the composition of the samples in some way.

In the previous work, typical binary system Cu_x_Zr_100-x_ thin film metallic glasses have been shown to have a high GFA in a wide range of compositions and great properties. For example, an enhanced elastic modulus (>109 GPa) with a good homogenous deformation is present in the Cu_50_Zr_50_ and Cu_64_Zr_36_ thin film metallic glass. Nevertheless, the corresponding hardness values (2.9 and 3.9 GPa) remain lower than that in Cu_50_Zr_50_ and Cu_64_Zr_36_ metallic glass ribbons (6.1 and 7.5 GPa) [32]. The addition of Al can improve not only the GFA but also thermal stability and especially mechanical property in Cu–Zr–Al metallic glass system [33]. Furthermore, with the addition of Al, the three empirical rules, which can improve the glass-forming ability and thermal stability of supercooled liquid, have been achieved [34,35]. Hence, in this work, we aim to study the possibility of fabricating a new Cu–Zr–Al ternary system thin film metallic glasses with various compositions and thicknesses deposited by DC magnetron sputtering to investigate their microstructure and properties. The composition and thickness of the thin film were controlled by the deposition powers. The corrosion and mechanical properties of as-deposited Cu–Zr–Al thin film, as well as the dependence of these properties on deposition conditions, were also studied.

## 2. Experimental

**Figure 1:**
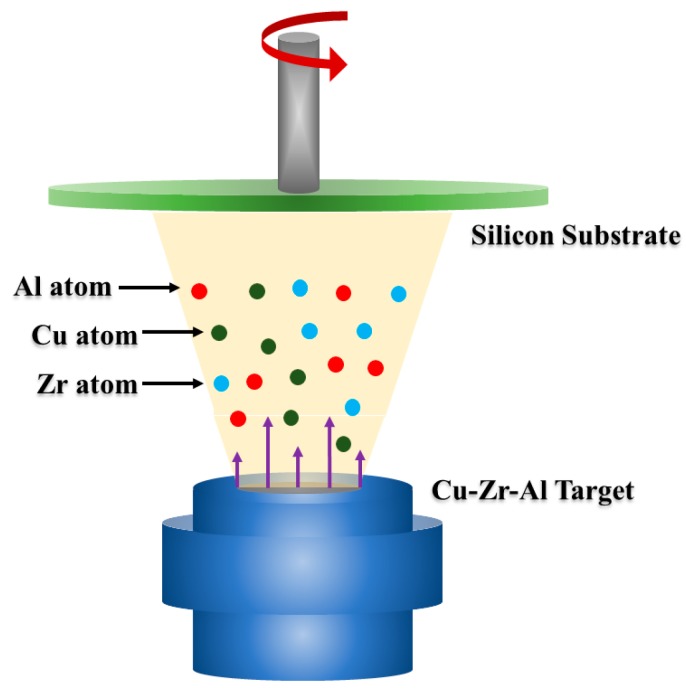
Schematics representation of Cu–Zr–Al thin film metallic glass deposited by magnetron sputtering.

The Cu–Zr–Al ternary alloy thin films were fabricated by DC magnetron sputtering (FJL-560, SKY, Shenyang, China) at room temperature. The schematic of DC magnetron sputtering process is shown in Figure 1. The target disk with a nominal composition of Cu_47.5_Zr_45.1_Al_7.4_ (at. %) was used as sputtering target, the target disk with a dimension of Φ 60 mm× 3 mm was prepared by vacuum hot pressing mixture powders of Cu, Zr, and Al at 1100 °C under 40 MPa, which was fixed at the bottom of the vacuum chamber. The substrate material used in this study is silicon wafer. The size of substrates is 10 mm × 10 mm. The substrates were installed on the rotation device right opposite to the target with a distance of 60 mm (as shown in Figure 1); the substrates were mirror polished and ultrasonically cleaned in acetone and alcohol each for 20 min. During the preliminary work, the deposition chamber should be evacuated to less than 10^−4^ Pa. After the 5 min plasma cleaning, high-purity Ar (99.99% purity) with a 30 sccm flow rate was gathered into the chamber to maintain the deposition pressure at 1.0 Pa. The sample rotation speed was constant at 8 rpm to make the Cu–Zr–Al thin film as uniform as possible, and the deposition time for each sample was 30 min. The specific deposition parameters are listed in Table 1. The target powers range from 36 W to 96 W.

The surface morphology and cross-section microstructure of Cu–Zr–Al thin films were detected by scanning electron microscope (SEM, Quanta 250, FEI, Hillsboro, OR, USA). The cross-section was acquired by directly scratching the diamond cutter on the silicon substrate and then breaking the sample off. The phase structure analysis was systemically performed by a grazing incidence X-ray diffractometer (GIXRD, Bruker D8, Germany) system with a Cu *Ka* X-ray source (1.54 Å) operated at 45 kV and 40 mA. The grazing incident angle for the tests is 0.5°, and the 2θ range is from 10° to 80° with a 10°/min scanning rate. The compositions of the films were measured by energy dispersive spectroscopy (EDS). The cross-sectional TEM foils were fabricated using a focused ion beam (FIB, Scios 2 Hivac, FEI, Hillsboro, OR, USA) system. The structure of Cu–Zr–Al thin film was characterized by transmission electron microscopy (TEM, Talos F200X, FEI, Hillsboro, OR, USA).

The corrosion property of Cu–Zr–Al thin film metallic glasses was measured by a standard three-electrode cell system, which contains a platinum counter-electrode and a saturated calomel reference electrode on the electrochemical workstation (Reference 600, Gamry, PA, USA). The samples were exposed in 1 mol/L of saturated H_2_SO_4_ solution. Prior to the measurements, the thin film samples were cathodic polarized to −1.2 V for 360 s to remove the oxide film in the air. The examinations began after the open circuit potential (OCP) was stable, and the electrochemical impedance spectroscopy (EIS) was conducted at a frequency range from 10^5^ Hz to 10^−^^2^ Hz. The potentiodynamic polarization shifted from −0.5 to 2.0 V with a potential sweep rate of 0.5 mV/s in the electrolyte when the OCP was almost stable. Each result was based on tests repeated at least three times to decrease the inaccuracy. To compare the corrosion properties, electrochemical tests were also carried out under the same conditions on 304 stainless steel (SS).

The mechanical property of Cu–Zr–Al thin film metallic glasses was characterized by a series of nanoindentation tests on a Nanoindenter G200 (Nanoscience Instruments, AZ, USA) indenter. The parameters of each test were maintained the same, and the experiment temperature was set at 29 °C. Before the experiment was performed, the indenter tip was calibrated, and the thermal drift value was reduced below <0.05 nm/s. The loading rate was 0.5 mN/s, and the maximum load was 360 mN. In this test, the fused silica material was selected as a reference during calibration to obtain reliable results. Each batch contained at least three indents, each test was performed with 40 μm distance between the consecutive indents to ensure accuracy.

## 3. Results and Discussion

**Figure 2:**
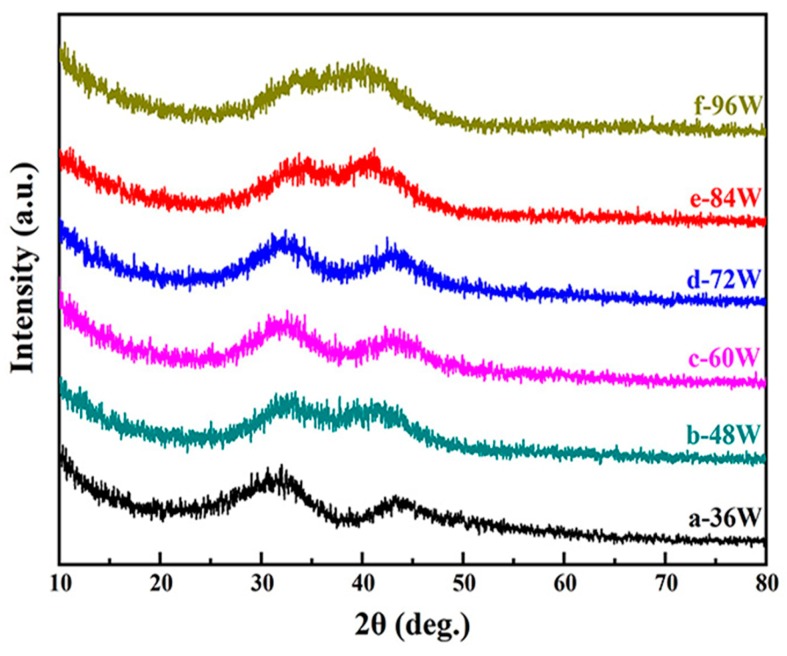
Grazing incidence X-ray diffractometer (GIXRD) patterns of Cu–Zr–Al thin film metallic glasses deposited at various sputtering powers.

Figure 2 shows the GIXRD patterns of Cu–Zr–Al thin films fabricated at different sputtering powers. In the previous studies, the XRD results of metallic glass materials often display one single broad diffuse peak, whereas in this result, two broad diffuse peaks were observed. As shown in the supplementary information (Figure S1), no crystalline phases were matched with the two broad diffuse peaks. The broad diffuse peaks suggest the amorphous structure of the Cu–Zr–Al thin films, whereas the two diffuse peaks may be caused by a phase separation phenomenon, which produces the two metallic glass phase structures in the Cu–Zr–Al thin films. The 2θ of two broad diffuse peaks were in the range of 30° to 45° with slight shifting, which may be caused by the composition changing in the metallic glasses [36]. The further verification of the metallic glass phases is discussed in the TEM results.

**Figure 3:**
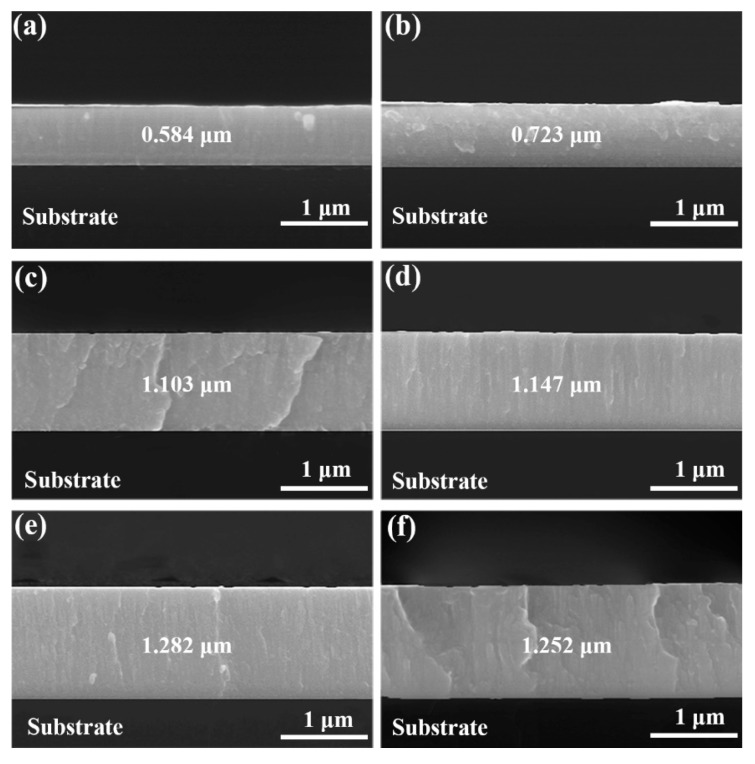
SEM cross-section images of Cu–Zr–Al thin film metallic glasses fabricated at different sputtering power. (**a**) 36 W, (**b**) 48 W, (**c**) 60 W, (**d**) 72 W, (**e**) 84 W, and (**f**) 96 W.

**Figure 4:**
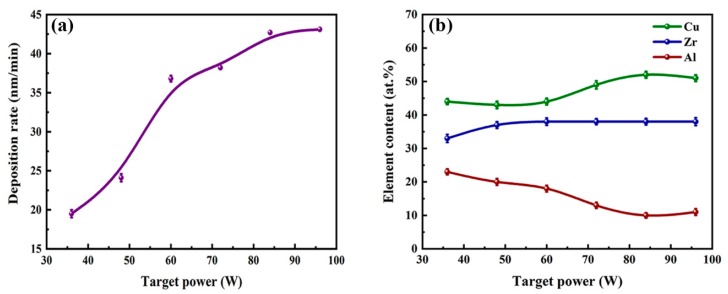
The deposition rate (**a**) and composition (**b**) of the as-deposited thin film as a function of the applied target power.

The surface morphologies of all as-deposited Cu–Zr–Al thin film metallic glasses deposited by magnetron sputtering are smooth and homogenous without apparent holes and cracks, which reflects the good quality of Cu–Zr–Al thin film metallic glasses (as shown in the supplementary materials, Figure S2). Figure 3 display the cross-section SEM images of Cu–Zr–Al thin film metallic glasses. The thin film metallic glass has a dense structure and adheres to the silicon substrate well. The thickness of each thin film metallic glass was measured from the cross-section SEM results (Table 1). As can be seen in Figure 3, the thickness is uniform through the whole thin film, and the magnetron sputtering power has a significant influence on the growth of the thin film metallic glass. The thickness is in the range of 0.584 to 1.282 μm, corresponding to a deposition rate of 19.5 and 42.7 nm/min, respectively. Figure 4a shows the deposition rate with the variation of sputtering power. The obvious increasing deposition rate was observed when the sputtering power was larger than 50 W; the higher power density enhanced the probability of ejection of atoms from the target, increasing the deposition rate [37]. When the sputtering power increased from 84 W to 96 W, the deposition rate slightly decreased. The possible mechanism of the deposition rate decrease is the re-sputtering of the film caused by the high energy ions [38]. The composition of each Cu–Zr–Al thin film was also analyzed by EDS. The average composition of the thin films is listed in Table 1. The composition of each thin film is different from that of the target, which is due to the different sputtering yield of Cu, Zr, and Al elements in the target at relatively low sputtering power [39]. With decreasing target powers, the Al content increases, whereas Cu and Zr contents decrease. The results indicate that the elemental composition of thin film metallic glasses can be precisely regulated by the appropriate target power.

**Figure 5:**
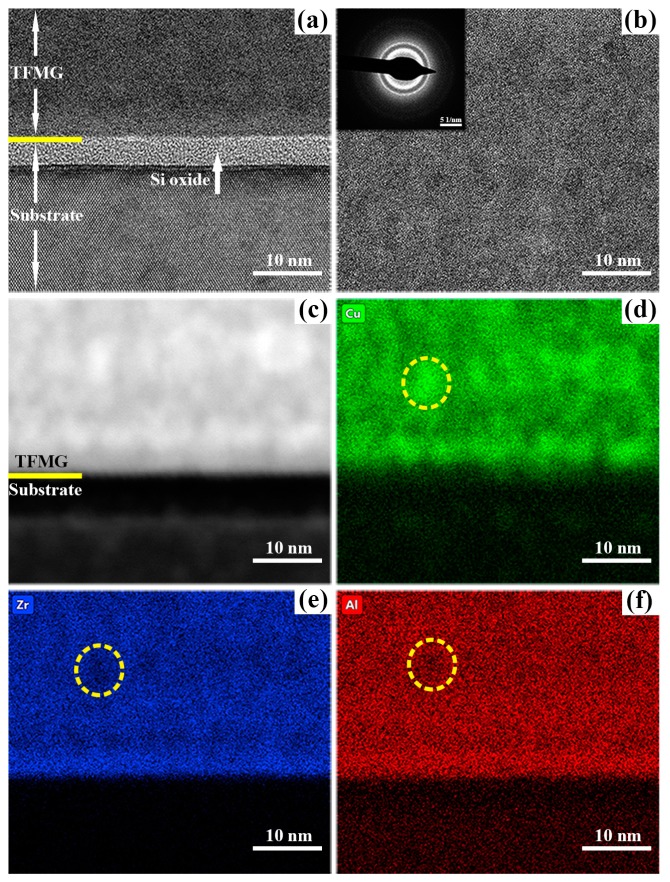
TEM results of the Cu–Zr–Al thin film metallic glass deposited at power of 72 W. (**a**) The high-resolution transmission electron microscope (HRTEM) image of the interface between the thin film and silicon substrate, (**b**) the HRTEM of Cu–Zr–Al thin film metallic glass (with insert of selected area electron diffraction, SAED pattern), (**c**) high-angle annular dark field (HAADF) image and (**d**–**f**) the EDS elemental mapping images of Cu, Zr, and Al element distribution.

The structure of as-deposited Cu–Zr–Al thin film metallic glass was also investigated by TEM. Figure 5 displays the TEM results of the Cu–ZrAl thin film metallic glass deposited at the power of 72 W, including the high-resolution transmission electron microscope (HRTEM) with the selected area electron diffraction (SAED) and EDS elemental mapping results of the detected field. Figure 5a shows the HRTEM image of an interface area of Cu–Zr–Al thin film metallic glass, which demonstrates an amorphous silicon oxide layer between the thin film metallic glass and the Si substrate. Figure 5b shows the SAED pattern and HRTEM image of the as-deposited Cu–Zr–Al thin film. The diffused diffraction ring and disordered atomic arrangement illustrate the metallic glass structure of the Cu–Zr–Al thin film. It is worth to mention that two broad and intense halos are observed in the SAED pattern. As shown in the supplementary information (Figure S3), besides these two diffuse halos, two other thin rings are also present. The radius of and corresponding d-spacing of four SAED rings were measured (Table S1). It can be concluded that the four rings present in the SAED pattern of Cu–Zr–Al thin film metallic glass deposited at power of 72 W do not belong to face centered cubic (FCC), body centered cubic (BCC), or any other crystalline phases. To further investigate the structure of Cu–Zr–Al thin film, the diffraction intensity profiles were obtained from a one-dimensional section of the SAED pattern in Figure 5b using the RDFTools software (Ver 1.1) [40]. Figure S4 in the supplementary information shows the SAED and XRD pattern with wave vector *Q* (*Q* = 4π sin*θ*/*λ*). The measured diagrams represent two broad peaks at *Q_1_* = 22.5 nm^−^^1^ and *Q_2_* = 30.0 nm^−^^1^. It has been reported that the amorphous hump of Cu–Zr binary amorphous alloy shifted to higher *Q* value by increasing the Cu content [31,41,42], while an amorphous hump with a lower *Q* was observed in Cu–Zr amorphous alloy with a higher Zr content [43]. The two broad peaks with different *Q* values indicate the presence of two Cu-rich and Zr-rich amorphous phases. The contrast of the HAADF image in Figure 5c indicates the elemental content variation in the Cu–Zr–Al thin film metallic glass. The elemental mapping images of Cu, Zr, and Al in Figure 5d–f also confirm the large compositional fluctuations on the nanometer scale, corresponding to the Cu- and Zr-rich amorphous phases. The Al content of the Zr-rich field is more than that in the Cu-rich field, which is attributed to the strong chemical affinity between Al and Zr [44]. The HAADF results of the 72 W Cu–Zr–Al thin film confirm the presence of two Cu- and Zr-rich amorphous phases. In previous investigations, in situ formed two metallic glass phases have been found in Zr–Cu–Ni–Al [3], Zr–Co–Al–Y [45], Cu–Zr–Al–Nb [46], La–Zr–Al–Cu–Ni [14], and Zr–Ce [47] alloys. The phase separation was observed in either the solidification or reheating process of those metallic glasses, in which the alloy was separated into two different glassy phases with different compositions. It is well known that the phase separation always occurs in the alloy systems whose heat of mixing value between two constituent elements are zero or positive [48]. However, the Cu–Zr, Zr–Al and Cu–Al pairs of Cu–Zr–Al thin film metallic glass all show negative heat of mixing (Cu–Zr: −23 kJ/mol, Zr–Al: −44 kJ/mol, Cu–Al: −1 kJ/mol) [49]. Meijering [50,51] proposed that a single regular solution could decompose into two different solutions even if the mixing enthalpies between the constituent elements were all negative. The phase separation in alloys with negative heat of mixing has been reported in Cu–Zr thin film [52] and Cu–Zr–Be metallic glass [53]. The mechanism of phase separation with negative heat of mixing is different from spinodal decomposition theory (the conventional phase separation caused by miscibility gap). Chen and Turnbull [54,55] proposed that the formation of a unique short-range order (SRO) in metallic glasses, which result in kinks or humps in the liquid/glass free energy curve (as shown in Figure S5), causing the thermodynamical phase separation [56].

**Figure 6:**
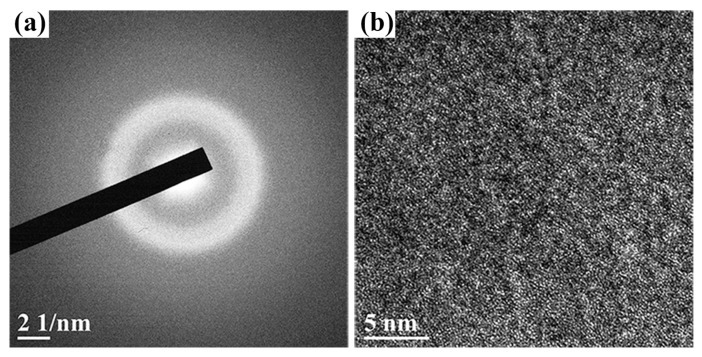
SAED pattern (**a**) HRTEM (**b**) image of the 96 W Cu–Zr–Al thin film metallic glass.

Figure 6a shows the SAED pattern of the Cu–Zr–Al thin film metallic glass deposited at the target power of 96 W. Unlike thin film deposited at the power of 72 W, only one broad and diffuse amorphous halo is observed, while short-range ordered clusters are present in the HRTEM image (Figure 6b). These results indicate that a tendency to nanocrystalline formation is present in thin film metallic glass deposited at target power of 96 W. The microstructure evolution in the 96 W sample is attributed to the annealing effect, arising from the substrate temperature rise induced by the high-energy Ar and metal ions bombardment on the film surface at high target power.

**Figure 7:**
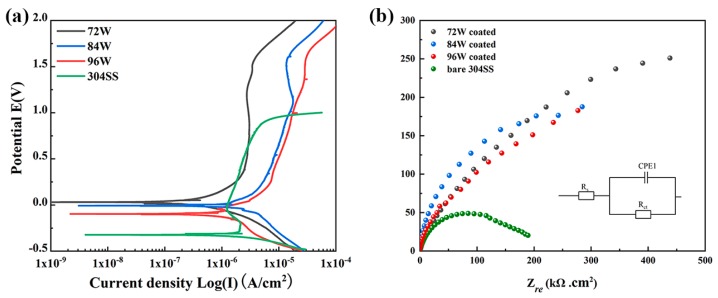
Potentiodynamic polarization (**a**) and electrochemical impedance spectra (EIS) (**b**) curves of Cu–Zr–Al thin film metallic glass and 304 stainless steel (as reference material) in 1 M in H_2_SO_4_

The corrosion resistance of the Cu–Zr–Al thin film metallic glass was tested by potentiodynamic polarization measurements. The typical potentiodynamic polarization curves for 72, 84, and 96 W Cu–Zr–Al thin film metallic glass samples and 304 stainless steel in 1 M H_2_SO_4_ exposed to air at 298 K are depicted in Figure 7a. It can be seen that the three potentiodynamic polarization curves of Cu–Zr–Al thin film metallic glasses present a similar profile. Based on these curves, the corresponding corrosion parameters, including corrosion potential (*E_corr_*), corrosion current density (*I_corr_*), passive current density (*I_pass_*), and pitting potential (*E_pit_*) are analyzed and summarized in Table 2. The *E_corr_* and *I_corr_* of 304 stainless steel are −323 mV and 1.05 μA·cm^−2^ in comparison with the coated Cu–Zr–Al thin film metallic glass samples. The *E_corr_* of 72 W sample (31.3 mV) is higher and its *I_corr_* (0.79 μA·cm^−2^) is lower than that in 304 stainless steel and Cu–Zr metallic glass coating [41], which indicates the improved corrosion resistance. The *I_corr_* of 84 and 96 W samples are 4.98 and 7.98 μA·cm^−2^ with corresponding *E_corr_* values of −3.79 and −117 mV, respectively. Furthermore, the pitting potential *E_corr_* of Cu–Zr–Al thin film metallic glasses (1.53–1.59 mV) is much higher than that of 304 stainless steel (−27.5 mV). The polarization curves of Cu–Zr–Al thin film metallic glass show a wide passive current density plateau, and the dissolution of passive films then causes the dramatic increase in current intensity at the pitting potential. The corrosion rate of 72 W thin film metallic glass (10.6 × 10^−3^ mmpy) is lower than that of Cu–Zr–Ti–Ni BMG (15.3 × 10^−3^ mmpy [57]) in 1 M in H_2_SO_4_ solution. The results indicate that the Cu–Zr–Al thin film metallic glass has excellent corrosion resistance to the H_2_SO_4_ acid. Figure 7b shows the EIS spectra of Cu–Zr–Al thin film metallic glasses. All Nyquist plots present only one capacitive loop. In general, the semicircle diameter has an association with the passive film, which is in accordance with the wide current density plateau in the potentiodynamic polarization curves. The EIS data is fitted by an equivalent circuit shown in the insert of Figure 7b, which consists of solution resistance (*R_s_*), thin film resistance (*R_ct_*), and the capacitance of the thin film (CPE). The fitting results based on the equivalent circuit are shown in Table 2. The value of *R_ct_* decreases rapidly with the increase of target power from 72 W to 96 W. The decreased *R_ct_* is due to the H_2_SO_4_ solution penetration into the thin film. Therefore, the results of corrosion tests demonstrate the improved corrosion of Cu–Zr–Al thin film metallic glasses.

**Figure 8:**
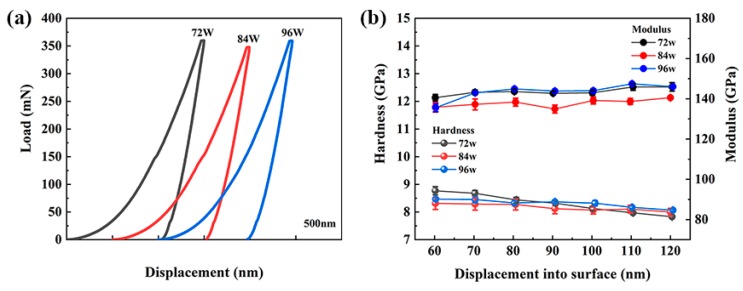
Loading–displacement curves (**a**), modulus and hardness variation of Cu–Zr–Al thin film metallic glasses (**b**).

The representative load–displacement (*P-h*) curves of Cu–Zr–Al thin film metallic glass samples (72, 84, and 96 W) are shown in Figure 8a. Each Cu–Zr–Al thin film metallic glass performs a homogenous deformation behavior for the reproducible and smooth characters in *P-h* curves. The appearance of a pop-in event in 72 and 84 W samples may relate with a shear band during the nanoindentation tests [58]. The variation trends of indentation hardness and modulus with pressed depths are shown in Figure 8b. The hardness was decreased with increasing pressed depth due to the indentation size effect of metallic glass [59]. The average hardness and the modulus of Cu–Zr–Al thin film metallic glasses can be gained by the stable situation at 50–120 nm of displacement. The depth selection is followed by the most important principle that the substrate effect must be avoided to ensure hardness and modulus values. Thus, displacement was selected at the end of 120 nm, which is less than 10% of the film thickness. The values of hardness and modulus are listed in Table 2. The hardness of 72, 84, and 96 W samples are 7.77, 8.18, and 8.17 GPa, whereas the modulus is noted as 145.92, 136.80, and 140.89 GPa. Both hardness and modulus values are much higher than the Cu–Zr binary system thin film metallic glasses and slightly higher than the Cu–Zr–Al bulk metallic glass [33]. It has been demonstrated that the sample size effect associated with the plastic deformation mechanism resulted in a higher hardness value of thin film [60]. The high elastic modulus in thin films may be related to their different short-range order or medium-range order (MRO) from rapidly quenched ribbon and bulk metallic glass samples [32].

## 4. Conclusions

A series of Cu–Zr–Al thin film metallic glasses with various compositions was successfully deposited by DC magnetron sputtering. The microstructure of the thin film metallic glasses is dense with neither voids nor cracks. The deposition rate and thin film compositions are directly related to the target power. The as-deposited thin film metallic glasses show an amorphous structure. The compositional fluctuations on the nanometer scale indicate the presence of two Cu- and Zr-rich amorphous phases. The Cu–Zr–Al thin film metallic glasses have excellent corrosion resistance in the sulfuric acid solution. The maximum hardness and elastic modulus of Cu–Zr–Al thin film metallic glasses deposited at a sputtering power of 72, 84, and 96 W can reach 8.18 and 145.92 GPa, respectively.

## Figures and Tables

**Table 1:** Target powers, thickness, deposition rate, and composition of Cu–Zr–Al thin film metallic glasses.

Samples	Power (W)	Thickness (μm)	Deposition Rate (nm/min)	Composition (at. %)
a	36	0.584	19.5	Cu_44_Zr_33_Al_23_
b	48	0.723	24.1	Cu_43_Zr_37_Al_20_
c	60	1.103	36.8	Cu_44_Zr_38_Al_18_
d	72	1.147	38.2	Cu_49_Zr_38_Al_13_
e	84	1.282	42.7	Cu_52_Zr_38_Al_10_
f	96	1.252	41.7	Cu_51_Zr_38_Al_11_

**Table 2:** The corrosion and mechanical properties of as-deposited Cu–Zr–Al thin film metallic glasses.

Samples		Corrosion Properties						Mechanical Properties		References
	Solution	*E_corr_* (mV)	*I_corr_* (μA·cm^−2^)	*I_pass_* (μA·cm^−2^)	*E_pit_* (mV)	Corrosion Rate (10^−3^ mmpy)	*R_ct_* (kΩ·cm^2^)	Hardness (GPa)	Modulus (GPa)	
72 W	1 M H_2_SO_4_	31.3	0.79	2.59	1.56	10.6	200.4	7.77	145.92	This work
84 W		−3.79	4.98	17.14	1.59	18.5	125.2	8.18	136.80	This work
96 W		−117	7.98	1.29	1.53	19.1	85.50	8.17	140.89	This work
304SS		−323	1.05	2.05	−27.5	25.1	49.45	−	−	This work
Cu-Zr coating	0.01 M H_2_SO_4_	−500	100	−	−	−	−	3.90	114.30	[32,41]
Cu_47_Zr_11_Ti_34_Ni_8_ BMG	1 M H_2_SO_4_	−427	−	−	−	15.3	−	−	−	[57]

## Supplementary Materials

The following is available online at https://www.mdpi.com/1996-1944/12/24/4147/s1, Figure S1: XRD pattern of Cu–Zr–Al metallic glass thin film deposited at 72 W. Figure S2: Surface morphology of Cu–Zr–Al thin film metallic glasses deposited at different sputtering power. Figure S3: SAED rings radius measurement for the Cu–Zr–Al metallic glass thin film deposited at 72 W. Figure S4: Electron (a) and XRD (b) patterns of the Cu–Zr–Al metallic glass thin film deposited at 72 W. Figure S5: A schematic diagram of phase separation for the system with negative heat of mixing. Table S1: SAED rings radius measurement results.
