# Supplementary material for: Fabrication, Corrosion, and Mechanical Properties of Magnetron Sputtered Cu–Zr–Al Metallic Glass Thin Film
Source: Materials (Basel). 2019 Dec 11;12(24):4147. doi: 10.3390/ma12244147 (PMC6947152; doi:10.3390/ma12244147)
Supplement: Supplementary file 1 [file materials-12-04147-s001.zip › materials-634613-SI.docx]

Supplementary Materials

Fabrication, Corrosion, and Mechanical Properties of Magnetron Sputtered Cu–Zr–Al Metallic Glass Thin Film

**Xianshun Wei ^1,2,^*, Chengxi Ying ^1^, Jing Wu ^3^, Haoran Jiang ^1^, Biao Yan ^1,2^ and Jun Shen ^1,4^**

^1^ School of Materials Science and Engineering, Tongji University, 4800 Caoan Road, Shanghai 201804, China; [1730620@tongji.edu.cn](mailto:1730620@tongji.edu.cn) (C.Y.); [1610434@tongji.edu.cn](mailto:1610434@tongji.edu.cn) (H.J.); [yan_biao@tongji.edu.cn](mailto:yan_biao@tongji.edu.cn) (B.Y.); [junshen@szu.edu.cn](mailto:junshen@szu.edu.cn) (J.S.)

^2^ Shanghai Key Laboratory for R&D and Application of Metallic Functional Materials, Tongji University, Shanghai 201804, China

^3^ SUSTech Cryo-EM Facility Center, Southern University of Science and Technology, Shenzhen 518055, China; [wujinguob@163.com](mailto:wujinguob@163.com)

^4^ College of Mechatronics and Control Engineering, Shenzhen University, Shenzhen 518060, China

***** Correspondence: [weixianshun@tongji.edu.cn](mailto:weixianshun@tongji.edu.cn)


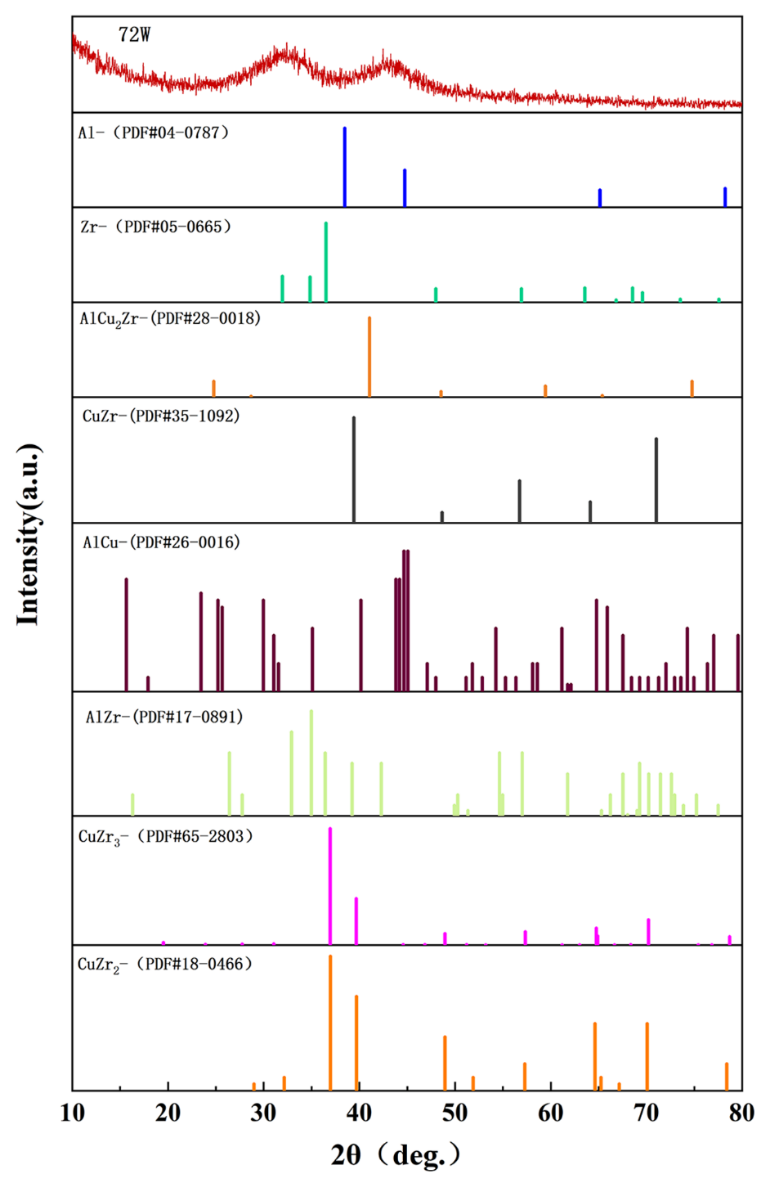


**Figure S1.** XRD pattern of Cu-Zr-Al metallic glass thin film deposited at 72W.


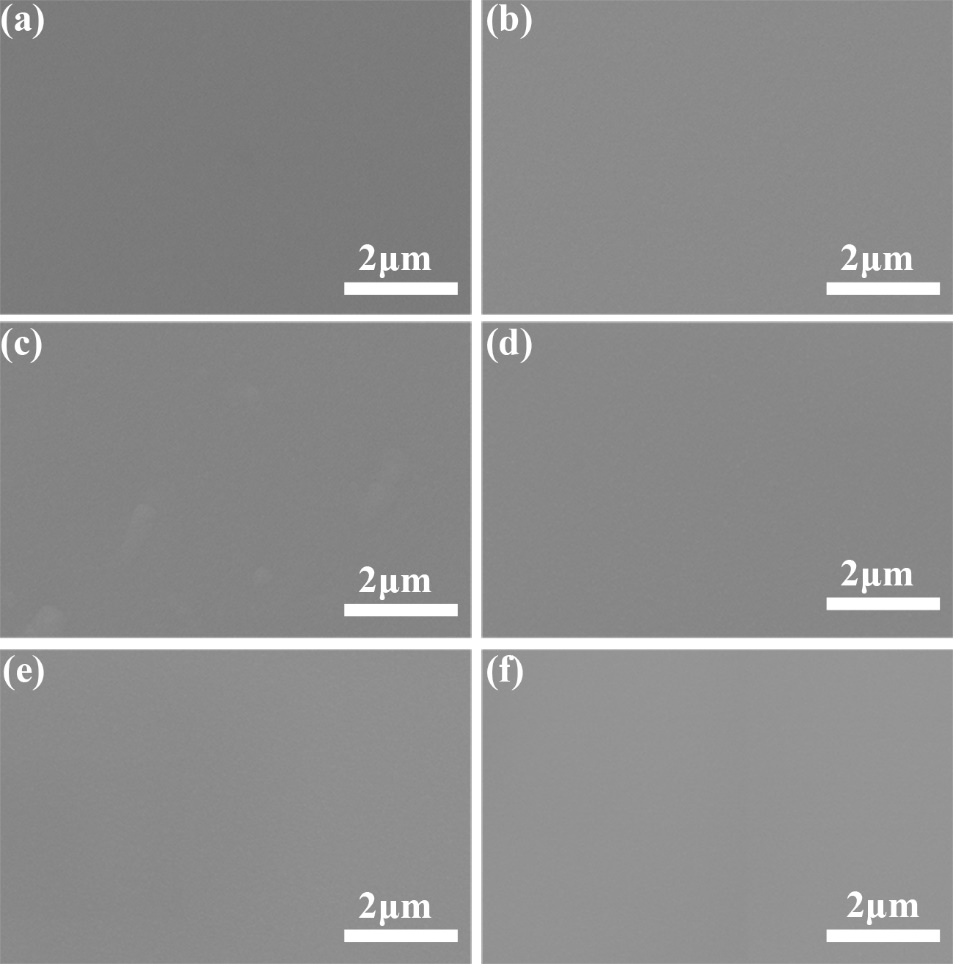


**Figure S2.** Surface morphology of Cu-Zr-Al thin film metallic glasses deposited at different sputtering power. (**a**) 36 W, (**b**) 48 W, (**c**) 60 W, (**d**) 72 W, (**e**) 84 W and (**f**) 96 W.


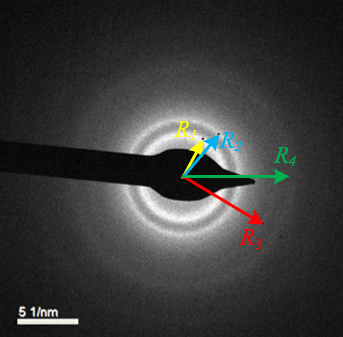


**Figure S3.** SAED rings radius measurement for the Cu-Zr-Al metallic glass thin film deposited at 72W.


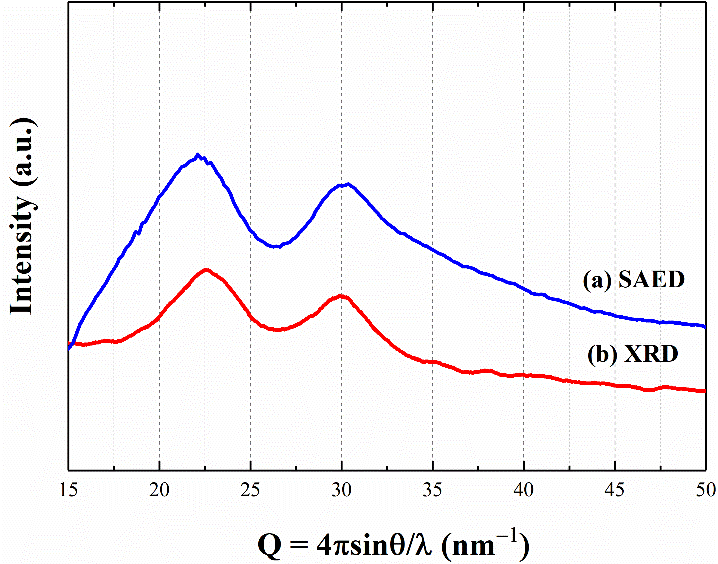


**Figure S4.** Electron (a) and XRD (b) patterns of the Cu-Zr-Al metallic glass thin film deposited at 72W.


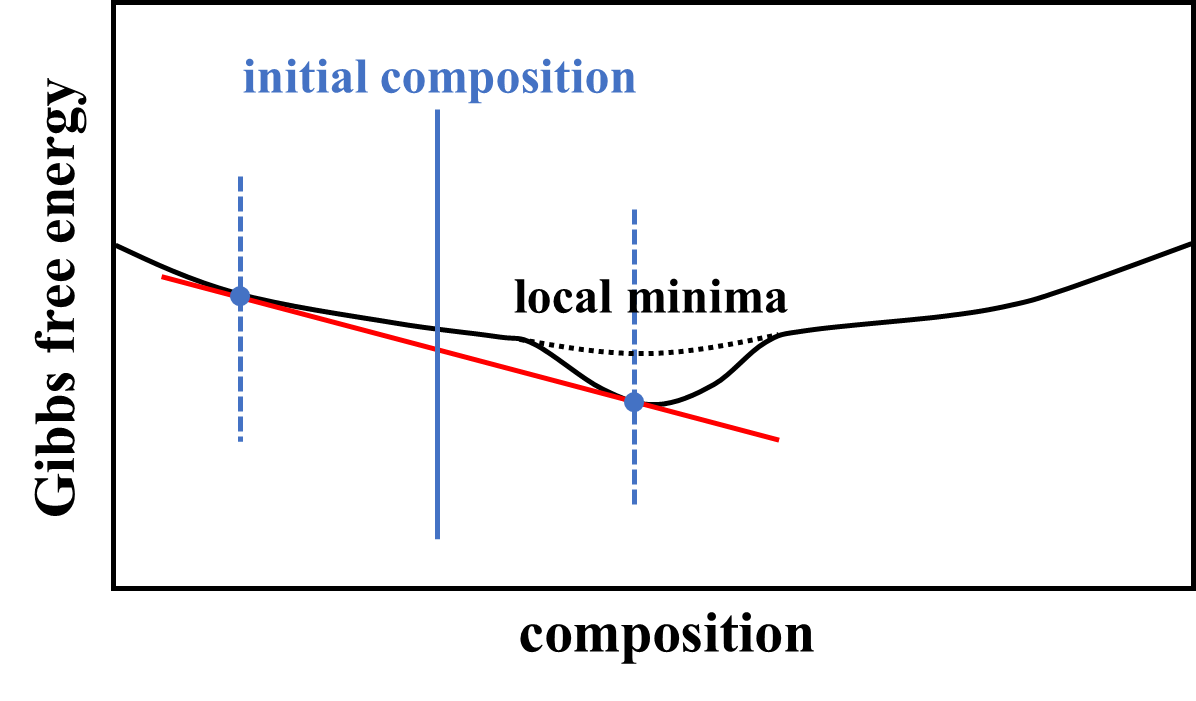


**Figure S5.** A schematic diagram of phase separation for the system with negative heat of mixing.

**Table S1.** SAED rings radius measurement results.

| **Ring Number** | **Radius (1/nm)** | **d-spacing** | **Cu-Zr-Al TFMG** | **FCC** | **BCC** |
| --- | --- | --- | --- | --- | --- |
|  |  | **(nm)** | ***R_i_^2^ / R_1_^2^*** | ***R_i_^2^ / R_1_^2^*** | ***R_i_^2^ / R_1_^2^*** |
| R1 | 3.622 | 0.2761 | 1 | 1 | 1 |
| R2 | 4.855 | 0.2060 | 1.79 | 1.33 | 2 |
| R3 | 7.705 | 0.1298 | 4.53 | 2.66 | 3 |
| R4 | 9.095 | 0.1099 | 6.30 | 3.67 | 4 |
